# Frequency-Amplitude Cross Interaction during Pulsatile Taste Delivery Using Gustometers

**DOI:** 10.3389/fnins.2016.00562

**Published:** 2016-12-15

**Authors:** Jean-Baptiste Thomazo, Adam Burbidge, Benjamin Le Révérend

**Affiliations:** Taste and Behavior Science, Nestlé Research CenterLausanne, Switzerland

**Keywords:** mixing, taste, gustometer, pulsatile delivery, transient perception

## Abstract

In this article, we numerically resolve the flow profiles of tastant concentration in the pipe of a gustometer used to deliver alternative pulses in concentration, which is a typical case of Taylor dispersion. Using this model, we can define the cases where the experimenter will deliver to the assessors a concentration profile which is significantly different from that intended. This can be simply assessed a priori using a scaling argument which involves calculating a dimensionless frequency. This is a function of the pulses frequency, the dimensions of the pipe and the flow rate used. We show that unless this parameter is taken into account, modifying the pulse frequency will modify the pulse amplitude. This design criterion is absent from the literature but we suggest this is important for designing such experiments.

## Introduction

In recent years, the idea of modulating chemosensations, in particular taste and aroma, using dynamically changing concentrations of chemicals, has emerged. This has been implemented in model foods such as gels (Stieger, 2011) and other types of solid foods such as bread (Noort et al., 2010, 2012) and bread crumbs (Konitzer et al., 2013; Pflaum et al., 2013), with various degrees of success and impact on taste perception. This approach is obviously an attractive route for sodium and sugar reduction in foods, which are particular areas of focus to both public health organizations (World Health Organization, 2012, 2015) and the food industry.

To establish the parameters of such dynamics, one often uses a liquid delivery system, named *gustometer*, to deliver dynamically changing concentrations (at a frequency ω) of chemicals to an assessor, rating either dynamically the taste intensity (TI) (Morris et al., 2009, 2010) or giving a global score to the sequence presented. Recently, Le Révérend et al. (2013) demonstrated that in mouth mixing of the different pulses was likely to occur, damping those dynamics at the taste cell/receptor level. Knowing how the boundary condition in concentration *C*_outlet_ varies with respect to time *t* when the liquid enters the assessor's mouth is important for the experimental and numerical approaches previously cited.

In this short communication, we show that not all gustometers are created equal with respect to *C*_outlet_(*t*), even though programming of the pumps and manifold appears to be the same. The dimensions of the pipe between the manifold or mixing cell and the assessor's mouth are of critical importance and yet often overlooked as design criteria.

## Approach

Gustometers are basically constructed from *n* pumps, whose outputs at flow rates $\dot{V_{i}}$ are mixed into a single flow channel, so that the flow rate received by the assessor is $\dot{V}=\overset{n}{\underset{i=1}{\sum }}\dot{V_{i}}$. The dimensions of pipe length, *L*, and radius, *R*, are of particular importance. *R* is supposed constant i.e., the tubing is stiff enough to accommodate variations of pressure during flow delivery without deforming. This is an important characteristic to ensure constant flow delivery. If the flow is steady and laminar (*Re* ≤ 2000)^1^, and the convection of the solute dominates over its diffusion (*Pe* ≫ 1)^2^, a phenomenon named *Taylor dispersion* occurs (Taylor, 1953), which practically increases the effective diffusion *D*_eff_ of the solute in the vehicle fluid following $D_{\text{eff}}/D\sim {\text{Pe}}^{2}$.

**Figure 1:**
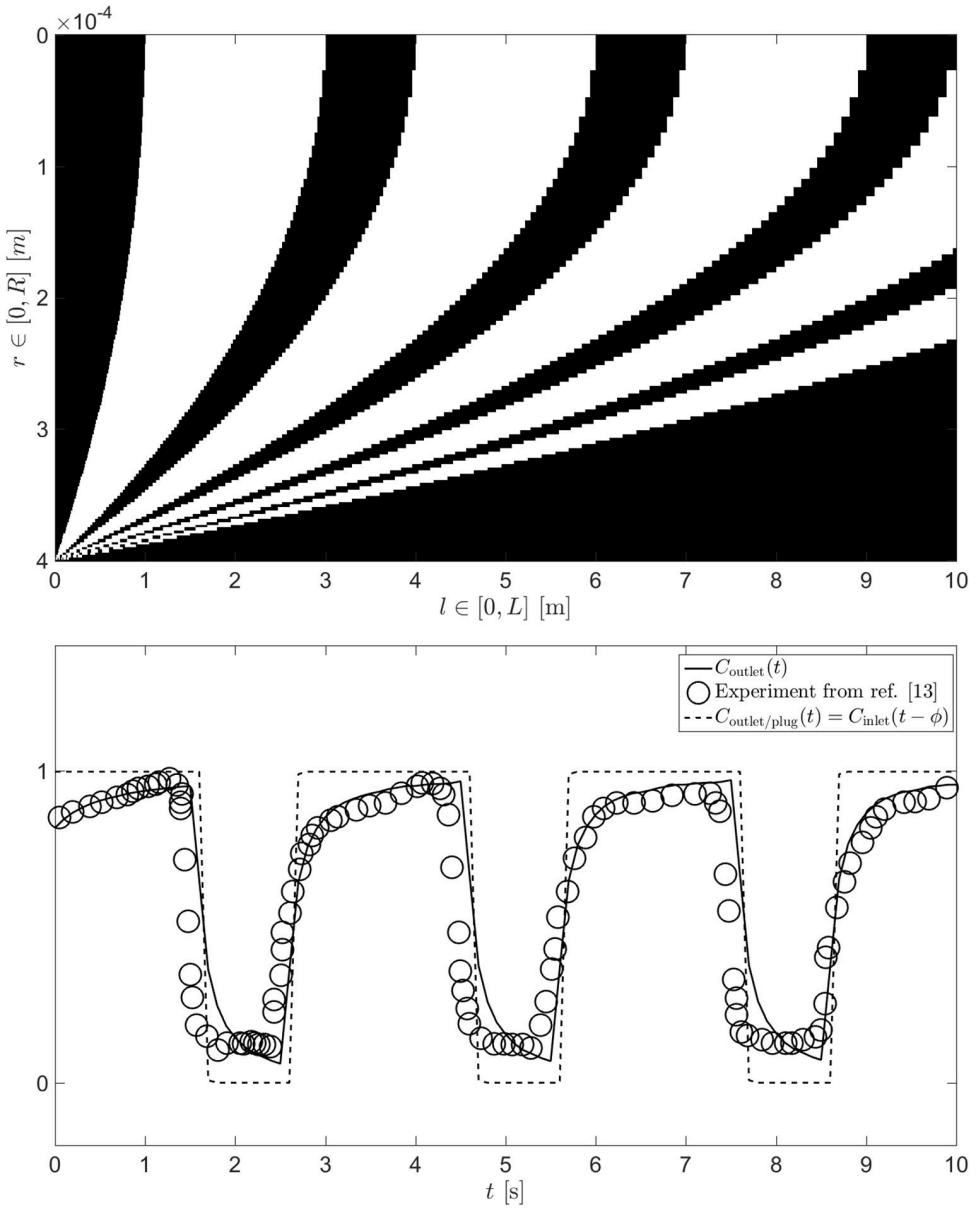
**Typical solution for a parabolic, laminar, diffusion free flow (***R*** = 0.4 mm, ***L*** = 10 m and $\dot{V}$ = 15 ml/min) when the inlet is subject to step changes in concentration (represented by the black and white colors) at a given frequency (ω = 1/3 Hz) [top]**. This results in an outlet concentration which is dephased by ϕ due to the length of the pipe and the average flow rate (as if the flow was plug-like, dashed line) and in addition smeared to form a saw tooth like signal (for the real parabolic case, solid line) **[bottom]**. In this case, taken from Burseg et al. (2010), the following parameters were used; *R* = 0.4 mm, *L* = 10 cm, ω = 1/3 Hz and $\dot{V}$ = 15 ml/min.

A simple way to represent this phenomenon is to imagine that, since the flow in the pipe is parabolic, fluid elements in the center of the pipe travel much faster than those at the periphery. The fluid at the center thus rapidly *catches up* the fluid at the periphery of the pipe from the previous pulse (Casadevall i Solvas and deMello, 2011), as shown in Figure 1. As the residence time of the fluid in the pipe increases, there is more time for dispersion and the more significant this phenomenon will become. If the pipe is long enough, a cross section of the pipe can contain fluid elements from many successive pulses, such that convolution of successive pulses of concentration occurs in the assessor's mouth (at the end of the pipe). If the Taylor dispersion is significant, a deviation from the commonly assumed (Bult et al., 2007) square wave function occurs, and the assessor experiences a sawtooth like signal, making the relevance of the experiment to model sharp changes in concentration flawed. In practice, as the pulse is smeared during the flow, the effective concentration span between two successive pulses will be reduced from *H* in the ideal case (i.e., the square wave at the start of the pipe) down to *h* < *H*. To measure this reduction in sensitivity we defined a dimensionless amplitude Δ = *h*/*H*. We chose to monitor this parameter Δ since the contrast between successive pulses is reported to be the source for the increase in perceived taste by the assessors (Burseg et al., 2010).

A classic approach to representing the behavior of a system in physics and engineering is to define dimensionless numbers that are products or ratios of physical dimensions, such that the number of parameters of importance is reduced to a kernel of parameters (Buckingham, 1915). Upon experimentally varying several physical dimensions, all experimental data should then collapse onto a single *master curve* when plotted as function of well chosen dimensionless parameters. In the light of the application of Taylor's work to *gustometers*, we propose to use the dimensionless frequency $\Omega =\omega \cdot L/\bar{\nu }=\omega \cdot L\cdot \pi \cdot R^{2}/\dot{V}$ (a Deborah number) which takes into account both the frequency of the pulses (ω) and the parameters of the equipment *L* and $\bar{\nu }$ (or $\dot{V}$ and *R*); and Δ as detailed above. Our objective with this model is to help the experimenter, who would like to know which pipe dimensions to use for a particular working flow rate and frequency.

## Solution and application to systems used in the literature

The *best case scenario* for the delivery of taste pulses is to assume a laminar, diffusion free flow, which thus minimizes mixing (see Figure 1). This can be solved using the analytical solution for the Hagen-Poiseuille flow in a pipe^3^ coupled with a change in concentration *C* of solute at the inlet of the pipe (*l* = 0). By weighting the radial flow rate^4^ with the local concentration *C*(*r, t*) of solute in the fluid lamina comprised between *r* and *r* + *dr*, we can then calculate *C*_outlet_(*t*)^5^. A typical solution for this problem (solved numerically with MATLAB ver. R2015b, on a 1000 × 1000 grid for pipe radius and length, respectively) is plotted at the bottom of Figure 1. One can see that the results deviate from the *plug flow* situation envisioned in the literature (Bult et al., 2007). The latter would lead to a simple dephasing of the outlet concentration compared to the inlet concentration by a phase lag $\phi =L/\bar{\nu }$, such that *C*_outlet/plug_(*t*) = *C*_inlet_(*t* − ϕ). Instead, the simulation predicts a sawtooth like profile, in excellent agreement with the outlet concentration that was measured by Burseg et al. (2010) (see Figure 1). Upon reaching steady state, one can define *h* = *max*(*C*_outlet_) − min(*C*_outlet_) and calculate Δ for a range of design parameters. The finite time taken to establish the steadiness of the pattern is an added complication that experimenters should also be aware of; detailed investigation of this is beyond the scope of this communication.

**Figure 2:**
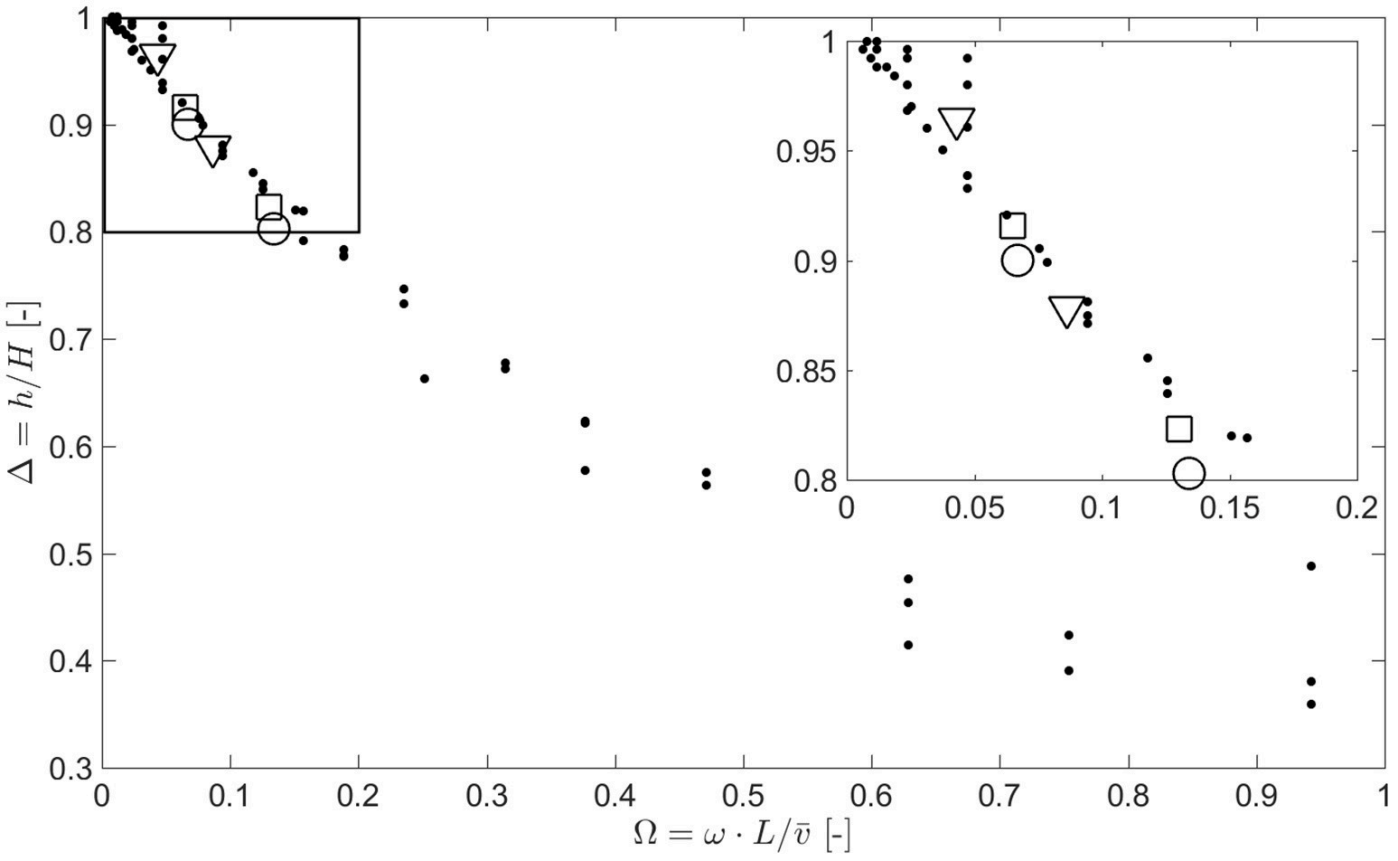
**Reduction of dynamic range of the gustometer Δ is controlled by the dimensionless frequency of the system $\Omega =\omega \cdot L/\bar{\nu }$**. Dispersion of the data points is due to numerical errors during discretization of time and space. Conditions from different systems available in the literature are plotted in open symbols: Burseg et al. (2010) (circles), Meiselman and Halpern (1973) (triangles), and Morris et al. (2009) (squares).

We carried out numerical simulations for a wide range of flow rates ($\dot{V}\in \left[0.2,100\right]\text{ml}/\text{min}$), pipe geometries (*L* ∈ [0.1, 5]m and *R* ∈ [0.1, 1]mm) and pulse frequencies (ω ∈ [0.2, 5]Hz) in order to map the experimental space of published gustometry. Given that we have identified two dimensionless quantities (Ω, Δ) that pertain to both the fluid/mass dynamics and the sensory readout of a gustometer experiment, we position the solutions and highlight different systems used in the literature on a (Ω, Δ) axis in Figure 2. As expected, all the computed solutions collapse on a single monotonous *master curve* suggesting that our scaling relationship is valid. Points are slightly scattered due to numerical errors during discretization of time and space.

To keep a good dynamic range (Δ > 0.9), one needs to have a system with a very low dimensionless frequency (Ω < 0.1). In practice it means that to have a good dynamic range to study very short pulses (high frequency ω), one would need either a very large flow rate ($\dot{V}$) or a very short and thin pipe. The study of such short pulses is of particular relevance for deciphering signaling proceeding from the peripheral and central integration cascade, which happens in a few hundreds of milliseconds (Tzieropoulos et al., 2013).

Having validated the scaling behavior with our simulations, we add to the graph simulations based on parameters used in three published studies (Meiselman and Halpern, 1973; Morris et al., 2009; Burseg et al., 2010). Divining exact parameters from such published studies is often difficult since potential modifications in either length *L* or radius *R* of the pipes are not always apparent to the reader. From the reviewed literature, we identified three main systems that have been developed to study those effects published by Meiselman and Halpern (1973), Hort and Hollowood (2004), and Bult et al. (2007). These systems have been used across many studies originating from the same laboratories or others, for example the setup used by Morris et al. (2009) is that developed by Hort and Hollowood (2004) and that used by Burseg et al. (2010) is that developed by Bult et al. (2007). Modifications of *L*, *R*, $\dot{V}$, and ω across those studies would strongly modify the dynamic range Δ of the system according to our simulations, and may not always be specified in the literature. In particular, the modification of the pulse frequency ω within a study has an important effect on the dynamic range Δ which in our opinion may prevent the authors from comparing sensory results and infer human somatosensory response as a function of ω.

Results from our simulations under the operating conditions of three studies are overlayed on the *master curve* in Figure 2. One can see that modifying ω from 0.25 to 0.5 Hz reduces the dynamic range of the experiment designed by Meiselman and Halpern (1973) from Δ = 0.96 to Δ = 0.88 (−8%) and from Δ = 0.92 to Δ = 0.82 (−11%) in the experiments of Morris et al. (2009). Similarly, modifying ω from 0.33 to 0.66 Hz reduces the dynamic range of the experiment designed by Burseg et al. (2010) from Δ = 0.90 to Δ = 0.80 (−11%).

## Conclusions

According to the results from our simulations, designing experiments to study the dynamics of chemical senses in mouth using a gustometer needs careful consideration of the fluid mechanics that will inevitably modify the outlet concentration compared to the inlet concentration. Studying the effect of pulse frequency on this perception requires careful validation of the steadiness of dynamic range at the outlet. This can be achieved by modifying pipe length, radius and flow rate between experiments to maintain a constant Ω, avoiding repercussions on the sensory readout. The simplest solution might be to work in a range where Ω tends to 0, for example in the absence of a pipe after the mixing chamber, such as that recently presented by Spector et al. (2015).

## Author contributions

All authors listed, have made substantial, direct and intellectual contribution to the work, and approved it for publication.

### Conflict of interest statement

AB and BL are employed by Nestlé Research Center and JT was employed by Nestlé Research Center at the time of the study. There have been no involvements that might raise the question of bias in the work reported or in the conclusions, implications, or opinions stated. The reviewer RH and handling Editor declared their shared affiliation, and the handling Editor states that the process nevertheless met the standards of a fair and objective review.

## Footnotes

^1^Reynolds number, $\text{Re}=2\cdot \bar{\nu }\cdot R\cdot \rho /\eta$, where $\bar{\nu }$ is the fluid average velocity, $\bar{\nu }=\dot{V}/\left(\pi \cdot R^{2}\right)$, ρ its density and η its dynamic viscosity. The solution is theoretically valid for any value of *Re*, as long as the flow is laminar. The value of 2000 is an empirical, commonly accepted, upper threshold for the laminar regime.

^2^Peclet number, $\text{Pe}=2\cdot R\cdot \bar{\nu }/D$, where *D* is the diffusion coefficient of this solute in the fluid vehicule.

^3^$\nu \left(r\right)=2\cdot \bar{\nu }\cdot \left(1-r^{2}/R^{2}\right),r\in \left[0,R\right]$ meaning that the velocity is null at the wall (*r* = *R*) and maximal along the axis of symmetry of the pipe (*r* = 0).

^4^$\dot{V}\left(r\right)\cdot dr=4\cdot \bar{\nu }\cdot \left(1-r^{2}/R^{2}\right)\cdot \pi \cdot r\cdot dr,r\in \left[0,R\right]$.

^5^$C_{\text{outlet}}\left(t\right)=\frac{\int_{0}^{R}C\left(r,t\right)\cdot \dot{V}\left(r\right)\cdot dr}{\int_{0}^{R}\dot{V}\left(r\right)\cdot dr}$.
